# Magnetite accelerates syntrophic acetate oxidation in methanogenic systems with high ammonia concentrations

**DOI:** 10.1111/1751-7915.13286

**Published:** 2018-06-12

**Authors:** Li Zhuang, Jinlian Ma, Zhen Yu, Yueqiang Wang, Jia Tang

**Affiliations:** ^1^ Guangdong Key Laboratory of Environmental Pollution and Health School of Environment Jinan University Guangzhou 510632 China; ^2^ Guangdong Key Laboratory of Agricultural Environment Pollution Integrated Control Guangdong Institute of Eco‐environmental & Technology Guangzhou 510650 China; ^3^ Energy and Environmental Engineering Institute Nanchang Institute of Technology Nanchang 330044 China

## Abstract

Ammonia accumulation is a major inhibitory substance causing anaerobic digestion upset and failure in CH_4_ production. At high ammonia levels, CH_4_ production through syntrophic acetate oxidization (SAO) pathways is more tolerant to ammonia toxicity than the acetoclastic methanogenesis pathway, but the low CH_4_ production rate through SAO constitutes the main reason for the low efficiency of energy recovery in anaerobic digesters treating ammonia‐rich substrates. In this study, we showed that acetate fermentation to CH_4_ and CO_2_ occurred through SAO pathway in the anaerobic reactors containing a high ammonia concentration (5.0 g l^−1^ NH_4_^+^–N), and the magnetite nanoparticles supplementation increased the CH_4_ production rates from acetate by 36–58%, compared with the anaerobic reactors without magnetite under the same ammonia level. The mechanism of facilitated methanogenesis was proposed to be the establishment of direct interspecies electron transfer (DIET) for SAO, in which magnetite facilitated DIET between syntrophic acetate oxidizing bacteria and methanogens. High‐throughput 16S rRNA gene sequencing analysis revealed that the bacterial *Geobacteraceae* and the archaeal *Methanosarcinaceae* and *Methanobacteriaceae* might be involved in magnetite‐mediated DIET for SAO and CH_4_ production. This study demonstrated that magnetite supplementation might provide an effective approach to accelerate CH_4_ production rates in the anaerobic reactors treating wastewater containing high ammonia.

## Introduction

The anaerobic digestion of organic wastes is regarded as one of important strategies for bio‐energy recovery. The concentration of ammonia has been found to be a vital factor affecting the performance and stability of CH_4_ production from anaerobic digestion (Rajagopal *et al*., 2013). High levels of ammonia, either present in protein‐rich wastes or accumulated during protein degradation, might cause a severe deterioration in the stability and performance of anaerobic digestion. In general, unstable performance or process failure occurs as the total ammonia nitrogen reaches 1500–7000 mg l^−1^ (Rajagopal *et al*., 2013), and the inhibiting ammonia concentrations depend on many factors such as the substrate, inocula, environmental conditions (temperature, pH) and acclimation periods (Chen *et al*., 2008). The direct inhibitory effects of high‐level ammonia on microbial activity, in particular, on acetate‐utilizing methanogens are considered as the primary cause of the decline in anaerobic digester performance and stability (Koster and Lettinga, 1984; Robbins *et al*., 1989).

In the anaerobic digester producing CH_4_, the energy sources for methanogens are limited to acetate, H_2_/CO_2_, CO, formate, methanol, ethanol, isopropanol, methylamines and methysulfides (Conrad, 2007; Borrel *et al*., 2011), and acetate is a very important precursor for methanogens producing CH_4_. Acetate degradation to CH_4_ can occur either via acetoclastic methanogenesis, in which acetate is cleaved into CH_4_ and CO_2_ by acetoclastic methanogens, or via syntrophic acetate oxidization (SAO), in which acetate is converted to H_2_/CO_2_ that is then reduced to CH_4_ by hydrogenotrophic methanogens (Westerholm *et al*., 2011). Although there are some conflicting findings about the sensitivity of acetoclastic and hydrogenotrophic methanogens to ammonia toxicity, major studies have found that hydrogenotrophic methanogens are more tolerant to ammonia stress than acetoclastic methanogens (Angelidaki and Ahring, 1993; Karakashev *et al*., 2005). Thus, SAO is frequently detected as the dominant acetate degradation pathway in anaerobic digester treating wastes containing high concentrations of ammonia (Karakashev *et al*., 2006; Li *et al*., 2014).

To alleviate the ammonia toxicity effect or to efficiently digest ammonia‐rich waste in anaerobic digesters, researchers have attempted the strategies of the bioaugmentation/accumulation of ammonia‐tolerant microorganisms, especially those involved in SAO metabolism (Chen *et al*., 2008; Fotidis *et al*., 2013a,b, 2014). Although SAO provides anaerobic digesters with the flexibility to respond to a high level of ammonia that leads to inhibitory activity for acetoclastic methanogens, methanogenesis via SAO is unfavourable in terms of CH_4_ production rate (Karakashev *et al*., 2006; Lee *et al*., 2015). Thus, how to facilitate CH_4_ production through SAO pathway is critical to improve the performance of anaerobic digestion with high levels of ammonia.

Conductive iron minerals (Kato *et al*., 2012; Viggi *et al*., 2014; Li *et al*., 2015; Yamada *et al*., 2015; Zhuang *et al*., 2015) and conductive carbon materials such as activated carbon (Liu *et al*., 2012), biochar (Chen *et al*., 2014a) and carbon cloth (Chen *et al*., 2014b) have been demonstrated to accelerate methanogenesis, in which these conductive materials mediate direct interspecies electron transfer (DIET) in syntrophic consortia. Kato *et al*. (2012) first reported that both magnetite and hematite stimulated methanogenesis from acetate through SAO in rice paddy soils, and DIET between syntrophic partners of *Geobacter* and *Methanosarcina* species occurred in the presence of conductive iron minerals. To the best of our knowledge, the possibility of magnetite supplementation to enhance the rates of CH_4_ production through SAO at high ammonia levels has not been previously investigated. Thus, the aim of this study was to examine magnetite supplementation as a potential method to trigger a faster syntrophic methanogenesis in the ammonia‐rich anaerobic digesters. CH_4_ production rates and microbial communities were characterized and compared in the magnetite‐free and magnetite‐supplemented anaerobic reactors with different ammonia concentrations. This allowed us to identify the mechanism of accelerated methanogenesis by magnetite supplementation under high ammonia concentrations.

## Results and discussion

### Influence of magnetite on acetate methanogenesis under different ammonia concentrations

**Figure 1:**
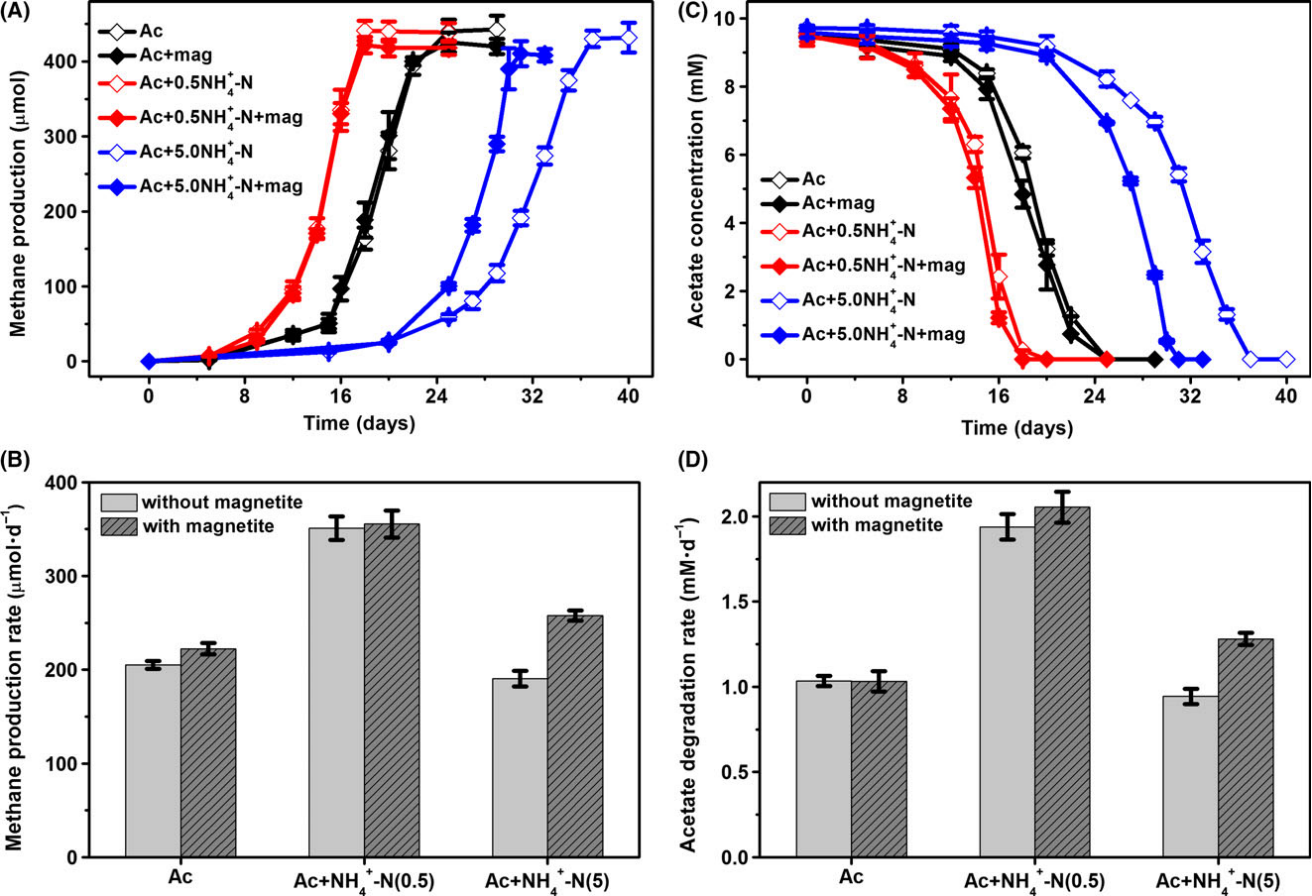
Methanogenesis from acetate under different ammonia concentrations in the presence or absence of magnetite. A. Time course of CH_4_ accumulation. B. Average CH_4_ production rates during the linear phase of metabolism estimated from the data in Fig. 1A. C. Time course of acetate degradation D. Average acetate degradation rates during the linear phase of metabolism estimated from the data in Fig. 1C. The error bars represent the standard deviations of three independent incubations.

In this study, the bioreactors were not previously acclimatized to ammonia. The accumulated CH_4_ production from the acetate‐fed incubations with different concentrations of NH_4_–N (0, 0.5 and 5.0 g l^−1^) is summarized in Fig. 1. After a short lag phase (6 days), the NH_4_–N‐free and 0.5 NH_4_–N bioreactors started to yield CH_4_ and proceeded to completion within 25 and 18 days respectively. The high concentration of ammonia (5.0 g l^−1^) significantly extended the lag phase of methanogenesis (20 days), and the magnetite‐supplemented and magnetite‐free incubations reached the plateau of CH_4_ production within 31 and 37 days respectively. Data demonstrated that the presence of magnetite did not substantially affect methanogenesis in both the NH_4_–N‐free and 0.5 NH_4_–N bioreactors. However, in the case of high ammonia concentration (5.0 g l^−1^), CH_4_ was generated more rapidly in the presence of magnetite than the unamended incubations. CH_4_ production rates during the linear phase of metabolism were estimated from the data in Fig. 1A and compared with respect to the supplementation of magnetite (Fig. 1B). With an ammonium concentration of 5.0 g l^−1^, magnetite stimulated methanogenesis from acetate with a rate (258 μmol day^−1^) that was 36% faster than that without magnetite (190 μmol day^−1^). During the second enrichment for the 5.0 NH_4_–N bioreactors, methanogenesis from acetate proceeded at higher rates without any lag phase, and the presence of magnetite enhanced the CH_4_ production rates by 58% (Fig. S1).

As shown in Fig. 1B, CH_4_ production rates in the 0.5 NH_4_–N bioreactors were higher than those in both the NH_4_–N‐free and 5.0 NH_4_–N bioreactors. The lower methanogenic activities in the NH_4_‐free bioreactors were likely due to the lack of a nitrogen source caused by the re‐suspension pretreatment of the sludge. Previous studies have evidenced that methanogenesis via SAO under high‐level ammonia concentrations is markedly slower than acetoclastic methanogenesis under low‐level ammonia concentrations (Karakashev *et al*., 2006; Lee *et al*., 2015), which is consistent with the lower CH_4_ production rates in the 5.0 NH_4_–N bioreactors relative to the 0.5 NH_4_–N bioreactors in this study. The second reason might be related to the direct toxicity of ammonia to methanogens (Chen *et al*., 2008; Westerholm *et al*., 2012), explaining the longer lag phase in the 5.0 NH_4_–N bioreactors.

As shown in Fig. 1C and D, the higher acetate degradation rates were mirrored by higher CH_4_ production rates. For all the bioreactors, the ultimate CH_4_ production was 86–94% of the value predicted from the stoichiometry of acetate degradation to CH_4_ (CH_3_COOH→CH_4_ + CO_2_) (Table 1), suggesting that methanogenesis was the predominant terminal electron‐accepting process in all of the acetate‐fed incubations. The rest fraction of acetate might be consumed by biomass growth (Siegrist *et al*., 2002) or alternative electron acceptors in the sludge. It is noticed that CH_4_ accumulation in the magnetite‐supplemented bioreactors was even lower, which might be resulted from acetate consumption by Fe(III) bioreduction. This was supported by Fe(II) production in the magnetite‐supplemented bioreactors (Fig. S2), and the more intensive Fe(III) reduction corresponded to the greater difference in CH_4_ recovery between the magnetite‐free and magnetite‐amended incubations (Table 1). The XRD measurements showed that, although Fe(III) reduction occurred, no new mineral product was formed, and no change occurred to magnetite particles before and after anaerobic incubation (Fig. S3).

**Table 1:** Stoichiometry of acetate methanogenesis (CH_3_COOH → CH_4_ + CO_2_)

	Bioreactors	Acetate utilized (mM)	Theoretical CH_4_ yield (μmol)	Observed CH_4_ yield (μmol)	Recovery (%)
1st enrichment	Ac	9.45	472.38	442.58 ± 18.45	94 ± 4
	Ac + magnetite	9.50	474.80	425.64 ± 12.93	90 ± 3
	Ac + 0.5NH_4_^+^–N	9.45	472.38	438.73 ± 12.61	93 ± 3
	Ac + 0.5NH_4_^+^–N + magnetite	9.50	474.80	418.42 ± 9.37	88 ± 2
	Ac + 5.0 NH_4_^+^–N	9.72	486.06	431.79 ± 20.07	89 ± 4
	Ac + 5.0NH_4_^+^–N + magnetite	9.58	479.23	410.24 ± 16.63	86 ± 3
2nd enrichment	Ac + 5.0 NH_4_^+^–N	3.01	150.50	136.54 ± 2.54	91 ± 2
	Ac + 5.0NH_4_^+^–N + magnetite	3.06	153.00	140.70 ± 3.43	92 ± 2

To further investigate the stimulatory effect of magnetite on methanogenesis, control bioreactors in the absence of acetate were conducted with the same inoculum and experimental conditions (Table S1). Data showed very low ‘endogenous’ CH_4_ accumulation in all the acetate‐free bioreactors, and methanogenesis in the presence of magnetite was even slower (Fig. S4). These results implied that (i) the re‐suspension pretreatment had almost removed the endogenous methanogenic substrate in the sludge; (ii) magnetite did not facilitate methanogenesis from acetate as an additional source of trace elements (e.g. iron ions); and (iii) the stimulatory effect of magnetite on methanogenesis under the high ammonia concentration was substrate dependent.

### Syntrophic acetate oxidation under a high level of ammonia

The high concentration of ammonia resulted in the deceleration of methanogenic processes in terms of the lag time and production rate, implying that the route of CH_4_ production in the 5.0 NH_4_–N bioreactors might be different from the 0.5 NH_4_–N bioreactors. Under high ammonia concentrations, SAO is more competitive than acetoclastic methanogenesis for CH_4_ production (Schnürer *et al*., 1999). SAO usually involves interspecies hydrogen transfer between acetate‐oxidizing bacteria and hydrogen‐utilizing methanogens, which maintaining a low hydrogen partial pressure (typically below 10^−5 ^atm) to keep anaerobic acetate oxidation energetically favourable (Stams and Plugge, 2009). Disrupting the delicate balance between syntrophic partners by increasing the concentration of hydrogen is an established method for detecting interspecies hydrogen transfer (Ahring and Westermann, 1988; Warikoo *et al*., 1996; Rakoczy *et al*., 2011; Viggi *et al*., 2014). Here, to identify the route of CH_4_ production under different ammonia concentrations and the influence of magnetite on the methanogenic pathway, the effects of hydrogen spiking on methanogenesis from acetate were investigated in both 0.5 NH_4_–N and 5.0 NH_4_–N bioreactors in the presence or absence of magnetite.

**Figure 2:**
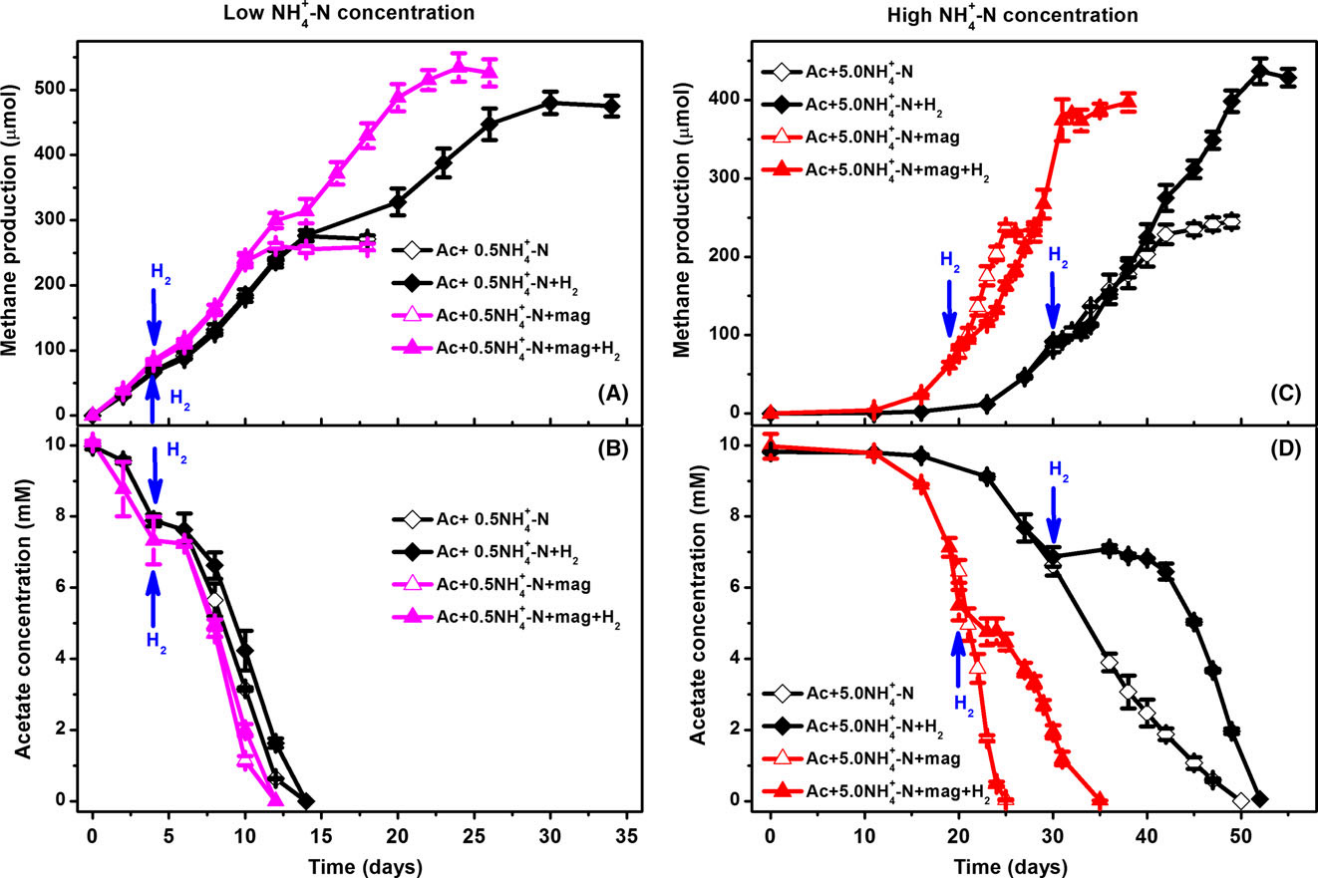
Methanogenesis from acetate under different ammonia concentrations in the presence of externally spiked H_2_. Time course of CH_4_ accumulation (A) and acetate degradation (B) under low ammonia concentrations; time course of CH_4_ accumulation (C) and acetate degradation (D) under high ammonia concentrations. The arrows denote the addition of H_2_. The error bars represent the standard deviations of three independent incubations.

When acetate was degrading to CH_4_, 20 ml of H_2_ was spiked into the headspace of the bottles, which were then shaken for proper mixing. As shown in Fig. 2A and B, the introduction of H_2_ did not affect the rates of acetate conversion to CH_4_ in the 0.5 NH_4_–N bioreactors with or without magnetite. During the early half of the incubation period, the kinetics of acetate degradation corresponded to the kinetics of CH_4_ production in all the 0.5 NH_4_–N bioreactors, suggesting that CH_4_ was generated from acetate. The presence of H_2_ increased the CH_4_ accumulation within the latter half of the incubation period, which was a result of the conversion of H_2_/CO_2_ to CH_4_. As expected, the effect of H_2_ spiking on acetate methanogenesis in the 5.0 NH_4_–N bioreactors was different with respect to magnetite supplementation (Fig. 2C and D). After H_2_ spiking, acetate degradation was temporarily inhibited for 10 days in the magnetite‐free bioreactors, while acetate degradation in the magnetite‐supplemented bioreactors was much less affected by H_2_ disruption. During the inhibition of acetate degradation, CH_4_ production was not affected and was compensated by the conversion of H_2_/CO_2_ to CH_4_, which gradually decreased the concentration of H_2_ and alleviated the inhibitory effect on acetate degradation. In summary, the results indicated that: (i) H_2_ spiking did not affect acetate degradation in the 0.5 NH_4_–N bioreactors but temporarily inhibited acetate degradation in the 5.0 NH_4_–N bioreactors, indicating the involvement of interspecies hydrogen transfer in the 5.0 NH_4_–N bioreactors. This provided an evidence for CH_4_ production via acetoclastic methanogenesis under low ammonia concentrations and via SAO under high ammonia concentrations. (ii) For the 5.0 NH_4_–N bioreactors, the process of acetate degradation in the bioreactors containing magnetite was much less affected by H_2_ disturbance than the magnetite‐free bioreactors. This implied that SAO, in the absence of magnetite, might highly depend on interspecies hydrogen transfer, whereas SAO in the presence of magnetite might not completely reply on interspecies hydrogen transfer.

**Figure 3:**
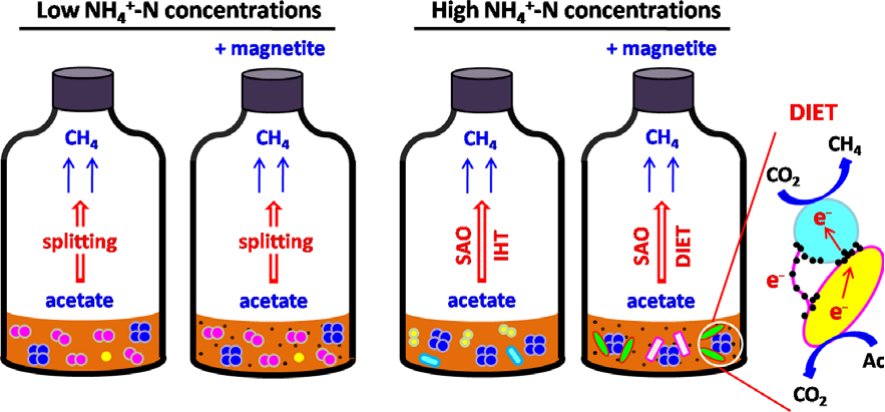
Models of the proposed methanogenic pathways under different ammonia concentrations in the presence or absence of magnetite. (DIET, direct interspecies electron transfer; SAO, syntrophic acetate oxidation; IHT, interspecies hydrogen transfer; Ac, acetate).

The alternative strategy for interspecies hydrogen transfer is DIET, in which two syntrophic partners exchange electrons via electrical interactions that may be facilitated by conductive pili and cytochromes (Summers *et al*., 2010) and abiotic conductive materials (Kato *et al*., 2012; Liu *et al*., 2012). Electrically conductive magnetite has been demonstrated to facilitate syntrophic methanogenesis from acetate, ethanol (Kato *et al*., 2012; Yamada *et al*., 2015), propionate (Viggi *et al*., 2014; Yamada *et al*., 2015), butyrate (Li *et al*., 2015) in rice paddy soils and anaerobic digesters. The mechanisms of facilitated methanogenesis are consistently proposed as DIET establishment between syntrophic partners through magnetite minerals, which can function as a surrogate for cytochrome OmcS that is important for DIET (Liu *et al*., 2015). The stimulatory effect of magnetite on methanogenic activity in the 5.0 NH_4_–N bioreactors was likely due to the presence of DIET promoting electron transfer between acetate‐oxidizing bacteria and methanogens, as depicted in Fig. 3.

### Microbial characterization

**Figure 4:**
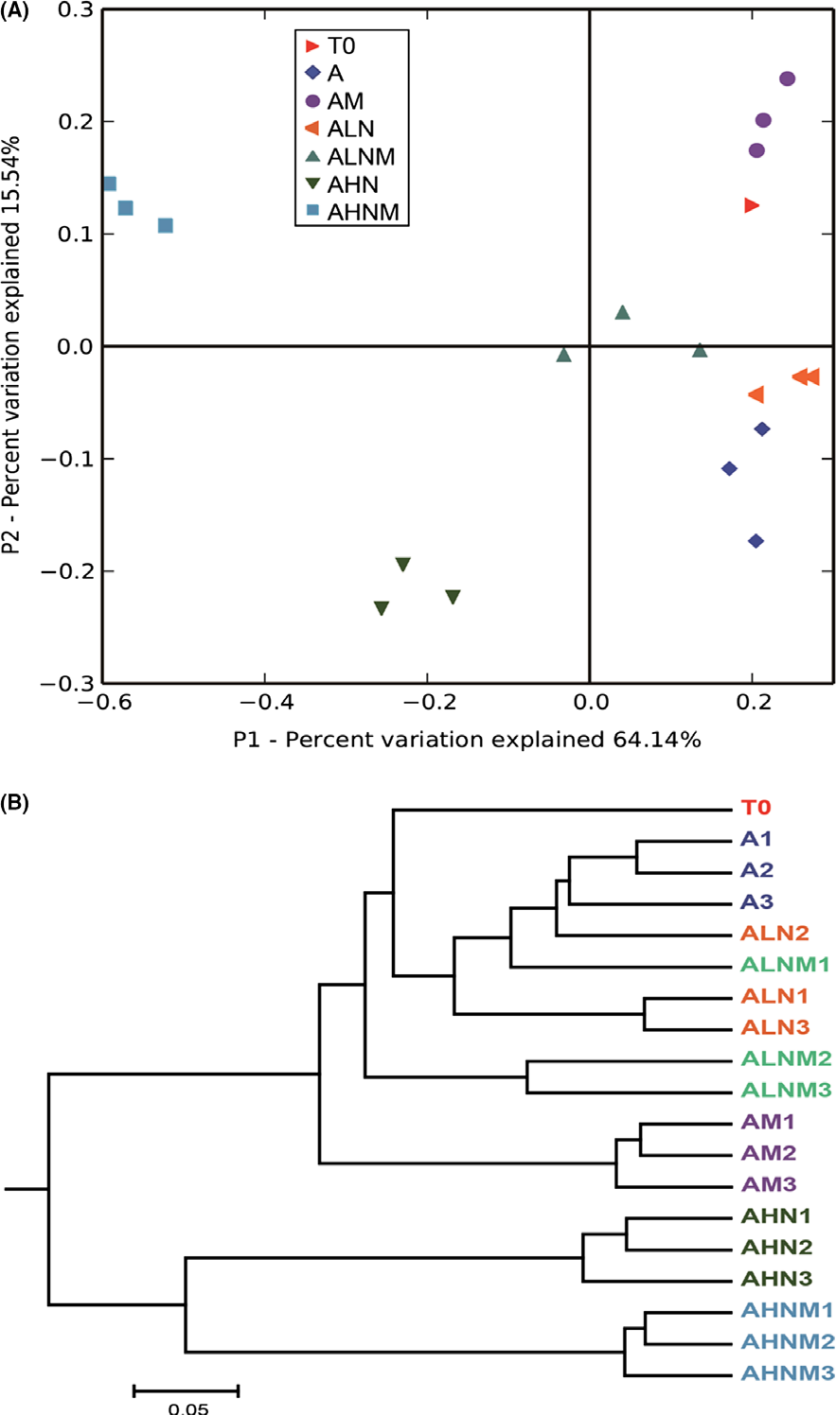
Principal coordinate analysis (PCoA) (A) and UPGMA tree (B) of the weighted UniFrac distance metric of samples obtained from methanogenic incubations. (T0: sludge inocula; A1–A3: triplicate of ammonia‐free incubations; AM1–AM3: triplicate of ammonia‐free incubations in the presence of magnetite; ALN1–ALN3: triplicate of 0.5 g l^−1^ NH_4_–N incubations; ALNM1–ALNM3: triplicate of 0.5 g l^−1^ NH_4_–N incubations in the presence of magnetite; AHN1–AHN3: triplicate of 5.0 g l^−1^ NH_4_–N incubations; AHNM1–AHNM3: triplicate of 5.0 g l^−1^ NH_4_–N incubations in the presence of magnetite).

To investigate microbial diversity and community structure in the bioreactors containing different ammonia concentrations, we sequenced the V4 hypervariable region of the 16S rRNA genes from all the bioreactors for bacteria and archaea using Illumina Miseq. An average of 13,550 ± 1103~19,068 ± 2057 high‐quality 16S rRNA gene sequences per sample with an average length of 307 bp were generated. The sequences were assigned to 1,410 ± 88~1,702 ± 54 OTUs per sample, with a distance limit of 0.03 (Table S3). The major bacterial populations in the bioreactors were associated with OTUs belonging to the phyla *Bacteroidetes*,* Firmicutes* and *Proteobacteria* (Fig. S5). Most of the archaeal OTUs in the bioreactors were assigned to the orders *Methanobacteriales*,* Methanomicrobiales* and *Methanosarcinales*. Distinct clusters of microbial communities are presented by PCoA in Fig. 4A, which showed that the microbial community in the high NH_4_–N bioreactors was significantly separated from the low NH_4_–N bioreactors by principle component 1. PCoA analyses revealed that the concentration of ammonia was an important effect on variation in the community structure; the presence of magnetite was also a determinant for community structure, especially for the 5.0 NH_4_–N bioreactors. Consistently, the UPGMA tree calculated from the weighted UniFrac distances showed that the microbial community in the reactors with high ammonia concentrations was grouped together and not related to other samples (Fig. 4B).

**Figure 5:**
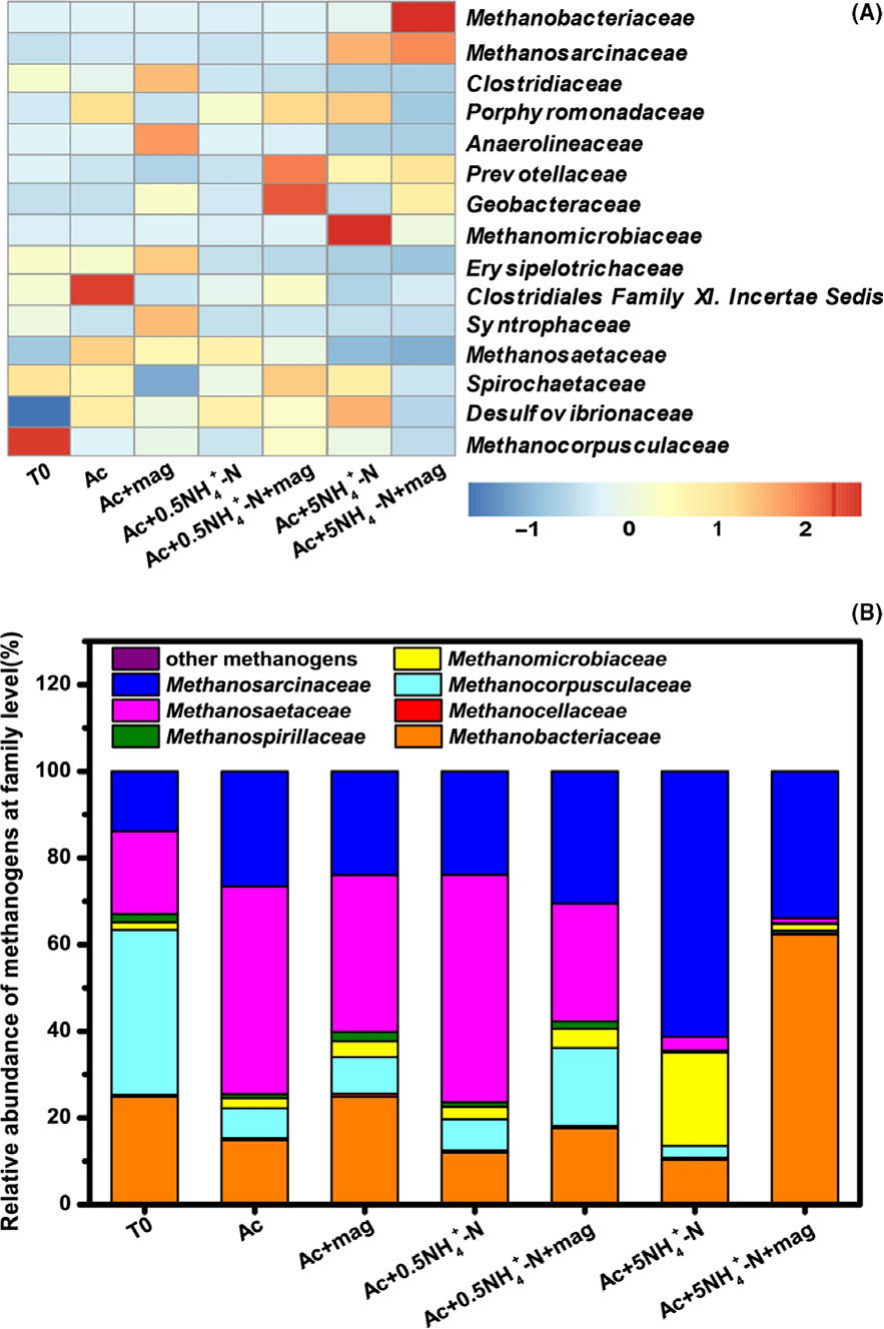
A. Heatmaps of the most abundant taxa (at the family level, at least >2% relative sequence abundance in one sample) for samples from methanogenic incubations under different ammonia concentrations in the presence or absence of magnetite. Blue denotes a low relative abundance across a taxon (row); red denotes a high relative abundance. The colour key for the *Z* score indicates correspondence between the blue‐red colouring and standard deviations from the mean abundance of each taxon. B. The fractions of known methanogens at the family level relative to the total number of methanogens in each incubation.

The family level characterization further illustrates the diversity of functional structures, and Fig. 5A shows a heat map of the most abundant bacterial and archaeal taxa with at least >2% relative sequence abundance in one sample. Evidently, the 5.0 NH_4_–N bioreactors were highly enriched in *Methanosarcinaceae* but deprived in *Methanosaetaceae*. This is consistent with previous studies on *Methanosaetaceae* vs. *Methanosarcinaceae* dominance in the case of high NH_4_‐N concentrations (Karakashev *et al*., 2005; Song *et al*., 2010), and the higher resistance of *Methanosarcina* against ammonia is likely due to its higher volume‐to‐surface ratio (Wiegant and Zeeman, 1986) and its more versatile spectrum of substrates utilization for CH_4_ production (Fotidis *et al*., 2013a,b). The hydrogenotrophic methanogens of *Methanobacteriaceae* and *Methanomicrobiaceae* were increased in the 5.0 NH_4_–N bioreactors with and without magnetite, respectively, in good agreement with previous finding that hydrogenotrophic methanogens are more tolerant to ammonia than acetate‐utilizing methanogens (Angelidaki and Ahring, 1993; Karakashev *et al*., 2005).

The influence of ammonia concentration on acetoclastic methanogens and hydrogenotrophic methanogens is vital to the route of CH_4_ production under different ammonia concentrations. Fig. 5B presents the fractions of known methanogens at a family level relative to the total methanogens in each bioreactor. The bioreactors with low ammonia concentrations were dominated by acetoclastic methanogens (*Methanosarcinaceae* and *Methanosaetaceae*). The fraction of *Methanosaetaceae* decreased dramatically in the 5.0 NH_4_–N bioreactors, providing a line of evidence for the SAO pathway in the 5.0 NH_4_–N bioreactors because the previous study had found a strong correlation between the absence of *Methanosaetaceae* and the involvement of the SAO pathway (Karakashev *et al*., 2006). These microbial analyses and the results derived from the H_2_ experiments supported that acetoclastic methanogenesis might be the dominant pathway at low ammonia concentrations, and that SAO methanogenesis was the prevailing pathway at high ammonia concentrations.

In the 5.0 NH_4_–N bioreactors, the acetate oxidizer of *Geobacteraceae* and methanogens of *Methanosarcinaceae* and *Methanobacteriaceae* were enriched by the supplementation of magnetite (Fig. 5A). *Geobacter* and *Methanosarcinaceae* have been demonstrated to be capable of performing DIET in iterative environmental and defined culture studies (Kato *et al*., 2012; Rotaru *et al*., 2014ba, b; Li *et al*., 2015). Although the syntrophic DIET function has not been demonstrated with *Methanobacteriaceae*, previous study found that the presence of *Methanobacteriaceae* coincided with the presence of magnetite and *Geobacteraceae* in magnetite‐facilitated DIET that stimulating syntrophic butyrate oxidation and CH_4_ production in paddy soil enrichment (Li *et al*., 2015). The possibility of *Methanobacteriaceae* capable of DIET function is very promising and deserves further investigations. *Geobacteraceae* were enriched in the magnetite‐supplemented bioreactors (Fig. 5A, Fig. S6), which might be a result of *Geobacteraceae* using Fe(III) from magnetite as electron acceptor (Fig. S2). However, this cannot support the relatively higher enrichment of *Geobacteraceae* in the 5.0 NH_4_–N bioreactors with the lowest extent of Fe(III) reduction, which implying the potential involvement of *Geobacteraceae* in methanogenesis from acetate. This further enhanced the possibility of methanogenesis through SAO by *Geobacteraceae* and methanogens of *Methanosarcinaceae* and *Methanobacteriaceae* in the presence of magnetite.

In sum, we studied the effect of magnetite supplementation on methanogenesis from acetate under low and high ammonia concentrations. Methanogenesis was dominant by acetoclastic methanogenesis under low ammonia level and SAO was more favourable under the high ammonia level. The supplementation of magnetite had no stimulatory effect on methanogenesis under low ammonia level, while accelerated methanogenesis via SAO by 36–58% under high ammonia level. The bacterial *Geobacteraceae* and the archaeal *Methanosarcinaceae* and *Methanobacteriaceae* were potentially involved in DIET‐mediated SAO and CH_4_ production, which was proposed as the mechanism of facilitated methanogenesis by the presence of magnetite.

## Experimental procedures

### Experimental set‐up

All of the experiments were performed using fresh anaerobic sludge as inoculum, which was collected from a full‐scale mesophilic wastewater treatment facility treating pig manure (Guangdong Province, China). Before experiments, approximately 1200 ml of sludge was centrifuged at 8000 rpm for 10 min, the supernatants were removed, and the centrifuged sludge was characterized in the general parameters (mg l^−1^): TOC:1110; COD_cr_: 3250; BOD_5_: 465; Organic matter: 2000; total nitrogen: 56; total P: 26. To prepare the bacterial inoculum, the solid sludge was re‐suspended in 135 ml of anaerobic basal medium as previously described (Hao *et al*., 2015). The details of the basal medium were provided in the Supporting Information.

Acetate methanogenesis experiments with different ammonia levels were carried out in 12 sets of bioreactors (each set has triplicate replications) as described in Table S1. NH_4_Cl was used as the ammonia source. Magnetite nanoparticles (Fe_3_O_4_, CAS number: 1317‐61‐9) were purchased from Sigma‐Aldrich Shanghai Trading Co. Ltd. (Shanghai, China), and the average diameter of particles is 50–100 nm by scanning electron microscope (SEM) measurements. The bioreactor experiments were conducted in 275‐ml anaerobic serum bottles containing 45 ml of basal medium, 5 ml of re‐suspended sludge, 10 mM acetate and 25 mM magnetite (as Fe atoms) if necessary. The effect of magnetite supplementation on acetate methanogenesis was investigated under low‐level (0.5 g l^−1^ NH_4_^+^–N) and high‐level ammonia (5.0 g l^−1^ NH_4_^+^–N). All of the anaerobic bottles were flushed with an 80%N_2_/20%CO_2_ (vol/vol) gas mixture for 60 min at a rate of 10 ml min^−1^ and were then sealed with Teflon^®^‐coated septa and aluminium crimp caps. The Eh, pH and conductivity of anaerobic bioreactors were measured upon the setting‐up (Table S2). To evaluate the influence of magnetite supplementation on the metabolism of the endogenous organic carbon, control incubations in the absence of acetate were also performed under the same experimental conditions. The bottles were incubated at a constant temperature of 37°C in the dark.

To verify the involvement of interspecies hydrogen transfer in the acetate conversion to CH_4_ (via SAO), additional batch experiments were conducted, in which H_2_ was added to the headspace of the bottles in the presence or absence of magnetite. For each bottle (125‐ml bottle containing 30 ml of medium), 20 ml of H_2_ was introduced using a sterile syringe when acetate was degrading to CH_4_. The concentrations of CH_4_ and acetate were periodically monitored after H_2_ addition.

### Chemical analysis

The CH_4_ concentrations in the gas samples were monitored using a GC9700 gas chromatograph (Techcomp Instruments, Shanghai, China) equipped with a flame ionization detector (FID). The temperatures of the inlet, oven and detector were 200, 80 and 350°C respectively. The injection volume was 200 μl, which was extracted from the headspace of the anaerobic bottles using a sterile syringe. The minimum detection limit of CH_4_ was 2 ppmv. After slightly vortex, three aliquots of liquid samples were extracted, and analysed for acetate, Fe(II) and ammonia. The concentrations of acetate were determined by HPLC (Shimadzu LC‐15C, Japan), equipped with a Wondasil C18 reverse‐phase column (250 mm by 4.6 mm, 5 μm pore size), and the lower detection limit was 1.0 mg l^−1^. The HCl‐extractable Fe(II) concentrations were determined via the ferrozine technique as previously described (Lovley and Phillips, 1986). Ammonium‐nitrogen was analysed by the Kjeldahl method, following American Public Health Association's Standard Methods (2005). All of the analyses were performed in triplicate, and the averages were presented along with the corresponding standard deviations (SD).

### DNA extraction and 16S rRNA gene sequencing

At the end of the incubation period (when CH_4_ production approached a plateau), the samples from the bioreactors were stored at –80°C until DNA extraction. The samples were collected by centrifugation (at 8000 *g* and 4°C for 10 min) and were then extracted using the PowerSoil™ DNA isolation kit (MO BIO Laboratories, USA) in accordance with the manufacturer's instructions. The DNA concentrations were determined using Qubit 2.0 Fluorometer (Invitrogen, NY, USA). Primer sets F515 (5′‐GTGCCAGCMGCCGCGGTAA‐3′) and R806 (5′‐GGACTACVSGGGTATCTAAT‐3′) were used to amplify the V4 hypervariable region of bacterial and archaeal 16S rRNA. Each DNA sample was amplified in triplicate, and the PCR details were provided in the Supporting Information. The PCR products were sent to Novogene (Beijing, China) for amplicon sequencing using an Illumina Miseq platform. The raw reads have been deposited in the sequence read archive section of NCBI with the following accession number: SRR4171267. Data analysis were performed following previously described methods (Caporaso *et al*., 2011) using the open source software package QIIME.

To assess the phylogenetic disparities between different microbial communities, principal coordinate analysis (PCoA) and UPGMA tree of weighted UniFrac distances were conducted and analysed with UniFrac software (Lozupone and Knight, 2005). Heat maps were created with the relative abundance data of the relevant taxa of the different samples and were normalized by calculating *Z*‐scores, which represent the relative abundance of taxa in a specific sample that differs from the mean relative abundance of that taxa in all samples normalized by standard deviations.

## Conflict of interest

None declared.

## Supporting information


**Fig. S1.** (a) Time course of CH_4_ accumulation; (b) average CH_4_ production rates during the linear phase of metabolism estimated from the data in Figure S4a for 5.0 g l^−1^ NH_4_‐N incubations during the second enrichment. The error bars represent the standard deviations of three independent incubations.
**Fig. S2.** The concentrations of HCl‐extractable Fe(II) in the bioreactors under different ammonia concentrations in the presence or absence of magnetite (CK denotes acetate‐free bioreactors; Ac denotes acetate). The error bars represent the standard deviations of three independent incubations.
**Fig. S3.** X‐ray diffraction spectrum of magnetite particle in the bioreactors before and after anaerobic incubation.
**Fig. S4.** Time course of CH_4_ accumulation in the acetate‐free bioreactors under different ammonia concentrations in the presence or absence of magnetite. The error bars represent the standard deviations of three independent incubations.
**Fig. S5.** The relative abundance of microbial community at phylum level according to 16S rRNA gene sequence in each incubation and initial sample.
**Fig. S6.** (a) The relative abundance of *Geobacteraceae* (%); (b) the gene copies of *Geobacteraceae* per gram wet sludge in the bioreactors under different ammonia concentrations in the presence or absence of magnetite. The error bars represent the standard deviations of three independent incubations.
**Table S1.** Set up of bioreactors.
**Table S2.** pH, conductivity, redox potential inside the initial bioreactors.
**Table S3.** Similarity‐based OTUs and species richness and diversity estimates.
