# Supplementary material for: Magnetite accelerates syntrophic acetate oxidation in methanogenic systems with high ammonia concentrations
Source: Microb Biotechnol. 2018 Jun 12;11(4):710–20. doi: 10.1111/1751-7915.13286 (PMC6011935; doi:10.1111/1751-7915.13286)
Supplement: Supplementary file 1 — Fig. S1. (a) Time course of CH4 accumulation; (b) average CH4 production rates during the linear phase of metabolism estimated from the data in Figure S4a for 5.0 g l−1 NH4‐N incubations during the second enrichment. The error bars represent the standard deviations of three independent incubations. Fig. S2. The concentrations of HCl‐extractable Fe(II) in the bioreactors under different ammonia concentrations in the presence or absence of magnetite (CK denotes acetate‐free bioreactors; Ac denotes acetate). The error bars represent the standard deviations of three independent incubations. Fig. S3. X‐ray diffraction spectrum of magnetite particle in the bioreactors before and after anaerobic incubation. Fig. S4. Time course of CH4 accumulation in the acetate‐free bioreactors under different ammonia concentrations in the presence or absence of magnetite. The error bars represent the standard deviations of three independent incubations. Fig. S5. The relative abundance of microbial community at phylum level according to 16S rRNA gene sequence in each incubation and initial sample. Fig. S6. (a) The relative abundance of Geobacteraceae (%); (b) the gene copies of Geobacteraceae per gram wet sludge in the bioreactors under different ammonia concentrations in the presence or absence of magnetite. The error bars represent the standard deviations of three independent incubations. Table S1. Set up of bioreactors. Table S2. pH, conductivity, redox potential inside the initial bioreactors. Table S3. Similarity‐based OTUs and species richness and diversity estimates. [file MBT2-11-710-s001.docx]

**Supplementary Materials**

**Magnetite accelerates syntrophic acetate oxidation in methanogenic systems with high ammonia concentrations**

**1 Supplementary data**

- 1. **Information for the reactor in the wastewater treatment facility**

HRT is 10 days, OLR is 1.5 Kg/(m^3^•d), and the ammonium concentration is 0.9 g/L.

- 1. **Component of the medium used in the batch experiments**

The anaerobic batch experiments were conducted in the serum bottles (275 mL) with 45 mL basal medium containing (per liter): 0.2 g MgCl_2_^.^6H_2_O, 0.1 g CaCl_2_, 0.2 g Na_2_S^.^9H_2_O, 2.77 g K_2_HPO_4_, 2.8 g KH_2_PO_4_, 5 mL trace element solution, and 2 mL vitamin solution. Yeast extract presented in the basal medium has been removed in our experiments to eliminate the possibility of CH_4_ production from yeast extract. The stock trace element solution contained (per liter): 1000 mg Na_2_-EDTA·2H_2_O, 300 mg CoCl_4_, 200 mg MnCl_2_·4H_2_O, 200 mg FeSO_4_·7H_2_O, 200 mg ZnCl_2_, 80 mg AlCl_3_·6H_2_O, 60 mg NaWo_4_·2H_2_O, 40 mg CuCl_2_·2H_2_O, 40 mg NiSO_4_·6H_2_O, 20 mg H_2_SeO_4_, 200 mg HBO_3_ and 200 mg NaMoO_4_·2H_2_O. The stock vitamin solution consisted of (per liter): 10 mg biotin, 50 mg Pyridoxin HCl, 25 mg Thiamine HCl, 25 mg D-Calsium pantothenate, 10 mg Floic acid, 25 mg Riboflavin, 25 mg Nicotinic acid, 25 mg P-aminobenzic acid and 0.5 mg vitamin B12. Sodium acetate was added as the methanogenic substrate to a concentration of 10 mM, and the initial pH was adjusted to 6.8 using 1 M HCl. NH_4_Cl was used as the ammonia source to regulate the ammonia concentration, and the low ammonia level and the high ammonia level were set as 0.5 g·L^-1^ and 5.00 g·L^-1^, respectively.

**1.3 16S rRNA gene sequencing method**

The PCR mixture (30 μL) contained 0.75 units Ex Taq DNA polymerase (TaKaRa, Dalian, China), 1× Ex Taq loading buffer (TaKaRa, Dalian, China), 0.2 mM dNTP mix (TaKaRa, Dalian, China), 0.2 µM of each primer, 100 ng template DNA. The ampliﬁcation conditions were as follows: initial denaturation at 94°C for 5 min; 35 cycles of denaturation at 94°C for 30 s, primer annealing at 53°C for 1 min, extension at 72°C for 1 min; and a final extension of 7 min at 72 °C.

**2 Supplementary figures and tables**

**2.1 Supplementary tables**

**Table S1** Set up of bioreactors

| **No. of bioreactor** |  | **Name of bioreactors** | **Conc. of NH_4_^+^-N (g/L)** | **Conc. of acetate (mM)** | **Magnetite (mM as Fe atoms)** |
| --- | --- | --- | --- | --- | --- |
| 1 | Control bioreactors without acetate supplementation | CK | 0 | 0 | 0 |
| 2 |  | CK+magnetite | 0 | 0 | 25 |
| 3 |  | 0.5 NH_4_^+^-N | 0.5 | 0 | 0 |
| 4 |  | 0.5 NH_4_^+^-N+magnetite | 0.5 | 0 | 25 |
| 5 |  | 5.0 NH_4_^+^-N | 5.0 | 0 | 0 |
| 6 |  | 5.0 NH_4_^+^-N+magnetite | 5.0 | 0 | 25 |
| 7 | Stimulated bioreactors with acetate supplementation | Ac | 0 | 10 | 0 |
| 8 |  | Ac+magnetite | 0 | 10 | 25 |
| 9 |  | Ac+0.5 NH_4_^+^-N | 0.5 | 10 | 0 |
| 10 |  | Ac+0.5 NH_4_^+^-N+magnetite | 0.5 | 10 | 25 |
| 11 |  | Ac+5.0 NH_4_^+^-N | 5.0 | 10 | 0 |
| 12 |  | Ac+5.0 NH_4_^+^-N+magnetite | 5.0 | 10 | 25 |

**Table S2** pH, conductivity, redox potential inside the initial bioreactors

| **Reactors** | **Eh (mV)** | **Conductivity (ms/cm)** | **pH** |
| --- | --- | --- | --- |
| 0.5 NH_4_-N | -102 | 12.37 | 6.58 |
| 0.5 NH_4_-N+magnetite | -96 | 12.46 | 6.56 |
| 5.0 NH_4_-N | -88 | over range of mS | 6.48 |
| 5.0 NH_4_-N+magnetite | -119 | over range of mS | 6.49 |

Note: the range of conductivity measurements is 0~20 ms/cm

**Table S3** Similarity-based OTUs and species richness and diversity estimates

| **Samples** | **Sequences** | **OTUs** | **Chao1** | **ACE** | **Shannon** | **Simpson** |
| --- | --- | --- | --- | --- | --- | --- |
| Day 0 | 14569 | 1802 | 2857 | 3031 | 7.81 | 0.982 |
| Ac | 19068±2057 | 1602±75 | 2325±29 | 2477±57 | 6.45±0.03 | 0.938±0.002 |
| Ac+magnetite | 13550±1103 | 1720±54 | 2737±20 | 2871±27 | 8.11±0.05 | 0.987±0.000 |
| Ac+0.5 NH_4_^+^-N | 15460±763 | 1418±54 | 2333±104 | 2469±95 | 6.00±0.29 | 0.883±0.028 |
| Ac+0.5 NH_4_^+^-N +magnetite | 14057±426 | 1624±82 | 2665±106 | 2796±91 | 7.55±0.23 | 0.978±0.007 |
| Ac+5.0 NH_4_^+^-N | 16172±1928 | 1410±88 | 2185±26 | 2275±46 | 6.94±0.08 | 0.966±0.003 |
| Ac+5.0 NH_4_^+^-N+magnetite | 18340±1422 | 1553±58 | 2293±2 | 2433±20 | 6.38±0.10 | 0.925±0.008 |

**2.2 Supplementary figures**

**Figure S1** (a) Time course of CH_4_ accumulation; (b) average CH_4_ production rates during the linear phase of metabolism estimated from the data in Figure S4a for 5.0 g·L^-1^ NH_4_-N incubations during the second enrichment. The error bars represent the standard deviations of three independent incubations.

**

**

**Figure S2** The concentrations of HCl-extractable Fe(II) in the bioreactors under different ammonia concentrations in the presence or absence of magnetite (CK denotes acetate-free bioreactors; Ac denotes acetate). The error bars represent the standard deviations of three independent incubations.





**Figure S3** X-ray diffraction spectrum of magnetite particle in the bioreactors before and after anaerobic incubation.**

**

**Figure S4** Time course of CH_4_ accumulation in the acetate-free bioreactors under different ammonia concentrations in the presence or absence of magnetite. The error bars represent the standard deviations of three independent incubations.

**

**

**Figure S5** The relative abundance of microbial community at phylum level according to 16S rRNA gene sequence in each incubation and initial sample.

**

**

**Figure S6** (a) The relative abundance of *Geobacteraceae* (%); (b) the gene copies of *Geobacteraceae* per gram wet sludge in the bioreactors under different ammonia concentrations in the presence or absence of magnetite. The error bars represent the standard deviations of three independent incubations.
